# Œdema of the Lungs—Intravenous Injection—Recovery

**Published:** 1880-07

**Authors:** 


### ŒDEMA OF THE LUNGS—INTRAVENOUS INJECTION—RECOVERY.

THE patient was a male, about 36 years of age. He was brought into the hospital under the influence of liquor and put in the cell to sleep off its effects. The next day the attention of the house physician was called to him as he seemed to be suffering greatly, and he was accordingly removed to a ward. On examination, it was discovered that there was considerable difficulty in breathing and that his feet and legs were much swollen and œdematous. He gave no history of previous illness—had not been sick before the beginning of his debauch. Had been drunk for several days before his entrance into the hospital. Physical examination gave no evidence of any abnormal condition of the heart, but loud, moist rales heard over the chest in front and behind indicated the presence of extensive œdema of the lungs. The patient was obliged to assume the sitting posture in bed as the dyspnœa increased. He coughed constantly and raised a thin, frothy sputum mixed with blood. His face was red and anxious; his pulse moderately full and rapid; his breathing was very labored, and his efforts to obtain air distressing. An examination of his urine failed to reveal the presence of albumen; its color was dark, its sp. gr. 1028. The dyspnœa being urgent and requiring active treatment, cups were applied over the whole chest and whisky was given freely, but with little apparent effect. The pulse became more feeble, the face livid, the extremities cold, and the whole body was covered with perspiration. The patient was every moment becoming more restless, turning his head from side to side, bending forward and tossing his arms about in vain efforts to breathe. Tinct. digitalis was then injected hypodermically mx. Oxygen was brought and mixed with air; it was inhaled by the patient, the tube being held to his mouth while he was directed to breathe through both mouth and nose. The effect of the digitalis was seen in a slight increase in the fullness of the pulse. The oxygen did not give the relief which was expected, and after a trial of twenty minutes was refused by the patient, who said it did no good. All the symptoms increasing, and there being added to them a frequent passage of small quantities of urine, a pill of croton oil, gt. j was given. This produced profuse liquid stools in about half an hour, with some slight improvement in the respiration. This improvement was, however, only temporary, and the dyspnœa soon became as marked as before. The patient was becoming more exhausted every moment, and the signs of carbonic acid poisoning were very evident, lividity of the face, apathy, disinclination to open the eyes or reply to questions, coldness of the extremities and face, while the pulse became more feeble and was scarcely perceptible at the wrist. As the treatment so far had seemed of but little avail, it was determined to inject aqua ammonia into the veins.

The cephalic vein was selected and exposed by a small incision, and with a hypodermic syringe fifteen minims each of aq. ammon. and aqua dest. were injected into it. The effects of the injection were felt in a few minutes, in the increased force of the pulse, while soon after the respiration became somewhat more easy. The patient was still, however, in much distress, and the dyspnœa subsided very gradually. During the following ten hours dry cups were again applied several times over the chest, and whisky and ammonia were given by the mouth. At the end of that time the breathing was much less difficult, though the moist rales could still be heard over the lower part of the chest in front and behind. Since then they have disappeared entirely, and the patient is in a fair way to recover. The absence of other causes for the œdema leads to the supposition that it was due to failure of the heart, resulting from exposure and alcoholism. No evidence of disease of the heart, lungs or kidneys could be discovered. But the depressing influence of large doses of alcohol long continued is well known, and it is evident in this case that the stimulant action being exhausted, a reaction and consequent enfeeblement occurred, which gave rise to the congestion of the lungs and œdema. This diagnosis was confirmed by the fact that further doses of alcohol, in the form whisky, failed to produce any effect on the heart. Bleeding, which was suggested, was happily avoided, and would probably have been fatal, as it would only have increased the exhaustion, without aiding the enfeebled organ. The injection of ammonia, which has been successful in many similar cases in the hospital, doubtless was the means of sustaining life here, as improvement began soon after. At any rate it is worth a trial in these cases.

